# Occupational fitness assessment in a schizophrenic patient applying for a nursing assistant position

**DOI:** 10.1192/j.eurpsy.2026.12174

**Published:** 2026-07-31

**Authors:** N. Gannoun, N. Belhadj, R. Nakhli, C. Sridi, M. Bouhoula, M. Makhloufi, I. Kacem, A. Aloui, J. Ben Khelifa, F. Chelly, I. Fki, M. Maoua

**Affiliations:** 1 Occupational Medicine Department, Sahloul University Hospital; 2 Faculty of Medicine of Sousse, University of Sousse; 3 sousse, Research laboratory (LR19SP03); 4 Occupational Medicine Department, Farhat Hached University Hospital of Sousse; 5 Clinical Psychology Unit, Occupational Medicine Department, Sahloul University Hospital, sousse, Tunisia

## Abstract

**Introduction:**

Schizophrenia is a chronic psychiatric disorder characterized by disturbances in thought, affect, and behavior. Its functional impact often raises concerns regarding occupational fitness and professional integration, particularly in healthcare-related jobs that involve relational responsibilities and emotional demands.

**(Case Presentation)**

We report the case of Ms. I.Z., 38 years old, followed since the age of 18 for paranoid schizophrenia and treated with haloperidol decanoate 300 mg every 21 days. She attended the occupational medicine service for a pre-employment examination as a nursing assistant. During the clinical interview, she presented with marked disturbances in thought and affect, including paranoid delusional ideas and incongruent laughter. A psychiatric assessment confirmed dissociative symptoms and superficial contact.

**Results:**

Despite the persistence of psychotic symptoms, professional integration was considered feasible under specific conditions. The nursing assistant position was judged compatible with adjustments such as fixed working hours, minimization of stress factors, and close medical monitoring to reduce relapse risk.

**Discussion**

This case highlights the pivotal role of occupational physicians in assessing job fitness in individuals with schizophrenia. Through collaboration with psychiatrists, a balance must be struck between patient safety, quality of care, and professional retention. Job adjustments and structured follow-up are essential to support integration while minimizing the risk of decompensation.

**Conclusion**

Occupational fitness assessment in patients with schizophrenia requires a multidisciplinary and individualized approach. Adapted working conditions and regular monitoring are key elements in promoting professional integration without compromising health or patient care.

**Disclosure of Interest:**

None Declared

